# Severe Rhabdomyolysis as an Unusual Presentation of Primary Human Immunodeficiency Virus Infection

**DOI:** 10.7759/cureus.3041

**Published:** 2018-07-24

**Authors:** Myint M Noe, Akriti G Jain, Sonia Shahid, Umair Majeed

**Affiliations:** 1 Internal Medicine Residency, Florida Hospital, Orlando, USA; 2 Internal Medicine, Karachi Medical and Dental College, Karachi , PAK; 3 Internal Medicine Residency, Florida Hospital-Orlando, Casselberry, USA

**Keywords:** rhabdomyolysis, acute retroviral syndrome, primary hiv infection

## Abstract

Rhabdomyolysis is characterized by muscle necrosis and leakage of toxic intracellular contents into the circulatory system. It is most commonly caused by trauma, physical exertion, drugs, toxins, and a variety of infections; only rarely is it associated with acute human immunodeficiency virus (HIV) infection alone. The severity of illness ranges from asymptomatic elevations in serum muscle enzymes to life-threatening electrolyte imbalances and acute kidney injury. High HIV viral load is associated with higher muscle breakdown that increases the incidence of severe acute kidney injury and sometimes the need for renal replacement therapy. The introduction of highly active antiretroviral therapy (HAART) revolutionized the treatment of HIV infection and increased the life expectancy of such patients by suppressing viral replication. Myopathy is one of the neuromuscular manifestations of HIV. It can occur either as a result of a complication of HIV itself or as a result of medicines used to control HIV. Muscle involvement of HIV infection ranges from asymptomatic muscle enzyme elevation to severe, HIV-associated polymyositis or pyomyositis. Here we report a case of acute retroviral syndrome presenting as severe non-traumatic rhabdomyolysis.

## Introduction

Rhabdomyolysis is characterized by muscle necrosis and leakage of toxic intracellular contents into the circulation. This clinically presents with muscular pain, weakness, and myoglobinuria. Increased myoglobin and creatine phosphokinase (CK) as a consequence of muscular cell death are the major laboratory findings [1]. It is most commonly caused by trauma, physical exertion, drugs, toxins, and a variety of infections. Rarely is it associated with acute human immunodeficiency virus (HIV) infection alone.

Primary HIV infection, or “acute retroviral syndrome,” is increasingly recognized as a clinical syndrome with a wide variety of presentations. Primary HIV infection appears to be symptomatic in most cases, but it is often unrecognized [2]. This syndrome typically occurs 2-6 weeks after exposure to HIV and it can present in a variety of ways: from a mild flu-like illness to a more severe constellation of complaints, including fever, fatigue, sore throat, anorexia, headache, myalgia, arthralgia, nausea, cervical adenopathy, diarrhea, or rash [2,3]. Photophobia, hepatosplenomegaly, and oral thrush have been reported, albeit with less frequency. Laboratory data often reveal thrombocytopenia, lymphopenia, and elevated hepatic transaminase levels [3].

Myopathy is a neuromuscular manifestation of HIV that can be caused by a complication of HIV itself but it could also result from the medicines used to control HIV. Muscle involvement of HIV infection ranges from asymptomatic muscle enzyme elevation, myalgia, and rhabdomyolysis to severe, HIV-associated polymyositis or pyomyositis [4].

The severity of rhabdomyolysis in HIV infection ranges from asymptomatic elevations in serum muscle enzymes to life-threatening electrolyte imbalances and acute kidney injury. High HIV viral load is associated with higher muscle breakdown, increasing the incidence of severe acute kidney injury and sometimes the need for renal replacement therapy [5]. The introduction of highly active antiretroviral therapy (HAART) revolutionized the treatment of HIV infection and increased patients’ life expectancy by suppressing the viral replication. Here we report a case of acute retroviral syndrome presenting as severe non-traumatic rhabdomyolysis.

## Case presentation

A 24-year-old African American male with no significant past medical history presented to the emergency department with a five-day history of severe weakness, generalized muscle pains, and decreased urine output. He denied any trauma, exertional activity, or prolonged immobilization. He also complained of a sore throat and mild diarrhea. He was taking naproxen 375 mg twice a day, prescribed for generalized pain four days before the admission. He denied any sick contacts or illicit drug use but admitted to being sexually active with multiple male partners. Physical examination was unremarkable except for oral thrush and reduced muscle strength in all extremities.

**Figure 1:**
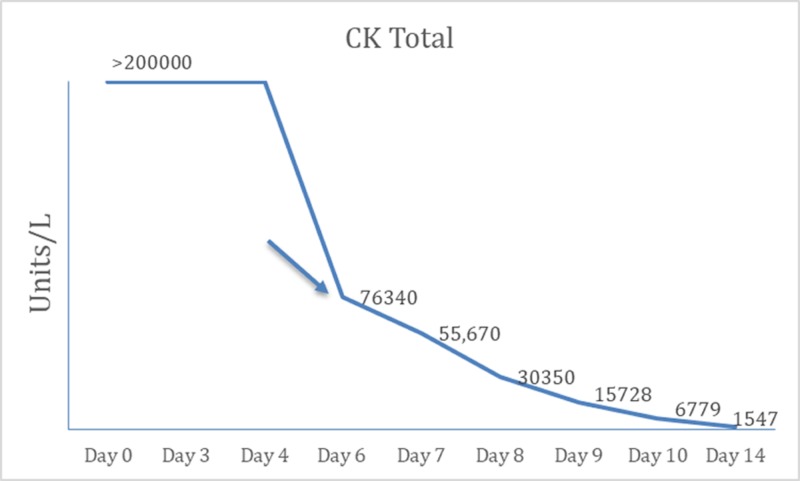
CK Total showing the exponential decline in CK after the patient was treated with HAART. Arrow denotes total CK after initiation of HAART on Day 6. CK: Creatine phosphokinase; HAART: Highly active antiretroviral therapy.

On initial laboratory investigations, serum creatinine, phosphorus, calcium, and uric acid levels were 5.7 mg/dL, 11.9 mg/dL, 5 mg/dL, and 13.6 mg/dL, respectively. Urine myoglobin was positive and creatine phosphokinase (CPK) was more than 200,000. The HIV-1/2 Antigen/Antibody screen was positive, Western blot was negative for both HIV 1 and 2 antibodies, HIV-1 ribonucleic acid polymerase chain reaction was detected above 10,000,000 copies/mL and CD4 count was 170 cells/mm^3^, all of which were consistent with early HIV infection before seroconversion. Serological tests for hepatitis B, hepatitis C, cytomegalovirus, Epstein-Barr virus, and urine drug screen were all negative. The patient developed anuria and fluid overload and was dialyzed through the right internal jugular catheter. He was started on a regimen of abacavir, lamivudine, ritonavir, and darunavir. About five days after the start of antiretroviral therapy, the patient’s symptoms and his CK level improved significantly (Figure 1). His urine output gradually increased and he was discharged home with scheduled outpatient dialysis.

## Discussion

Patients with early HIV infection can present with a variety of symptoms but rarely with severe rhabdomyolysis. Reportedly, rhabdomyolysis is increasingly recognized in HIV-infected population and can happen at any stage of HIV infection [6].

However, it is usually multifactorial, confounded by other potential causes. The concurrent risk factors of rhabdomyolysis include substance abuse, alcohol use, medication-related adverse effects such as the use of statins in combination with highly active antiretroviral therapy, opportunistic infections associated with HIV, or co-infection with other viruses including hepatitis C [7-9]. Integrase inhibitors approved for treatment of HIV infection and used in combination with other antiretroviral drugs have also been associated with rhabdomyolysis [6,10].

The incidence of rhabdomyolysis in the HIV population was 943 per 100,000 person-years. Data from Kaiser Permanente on HIV-positive individuals showed an incidence rate of 265 per 100,000 person-years [11]. A retrospective cohort study from Johns Hopkins Hospital studied patients’ characteristics and clinical outcomes of rhabdomyolysis in patients with HIV. The study showed that patients with a higher viral load were more likely to have an infectious etiology (p = 0.004); those with a suppressed viral load were more likely to have medications underlying the cause of their rhabdomyolysis (p < 0.001). The study also showed that rhabdomyolysis in HIV appears to be associated with an increase in mortality compared with the general population [5].

Chariot et al. attribute skeletal muscle involvement in HIV patients to immune-mediated mechanisms activated by HIV itself. The infiltration of inflammatory cells into the muscle leads to muscle injury and breakdown [12].

Patients with primary HIV infection can be asymptomatic or can present with a fever, sore throat, fatigue, and weight loss. Other presenting signs and symptoms may include lymphadenopathy, oral or genital ulcers, and aseptic meningitis [2]. Laboratory abnormalities can include lymphopenia, thrombocytopenia, and elevated hepatic transaminases [3]. Myalgias are common but rhabdomyolysis is not a typical presentation [2].

## Conclusions

As high HIV viral load is associated with higher CK level that increases the incidence of severe acute kidney injury, we believe that starting antiretroviral therapy early in this patient, during the acute phase of rhabdomyolysis, assisted in patient’s recovery from acute kidney injury and reduction of the muscle breakdown by suppressing the viral replication. In patients presenting with rhabdomyolysis, the possibility of acute HIV seroconversion should be considered, especially if they do not have other risk factors for rhabdomyolysis.

## References

[1] Giannoglou GD, Chatzizisis YS, Misirli G (2007). The syndrome of rhabdomyolysis: pathophysiology and diagnosis. Eur J Intern Med.

[2] Schacker T, Collier AC, Hughes J, Shea T, Corey L (1996). Clinical and epidemiologic features of primary HIV infection. Ann Intern Med.

[3] Kahn JO, Walker BD (1998). Acute human immunodeficiency virus type 1 infection. N Engl J Med.

[4] Chariot P, Gherardi R (1995). Myopathy and HIV infection. Curr Opin Rheumatol.

[5] Koubar SH, Estrella MM, Warrier R, Moore RD, Lucas GM, Atta MG, Fine DM (2017). Rhabdomyolysis in an HIV cohort: epidemiology, causes and outcomes. BMC Nephrol.

[6] Dori L, Buonomini AR, Viscione M, Sarmati L, Andreoni M (2010). A case of rhabdomyolysis associated with raltegravir use. AIDS.

[7] Ito H, Ito H, Nagano M, Nakano S, Shigeyoshi Y, Kusaka H (2005). In situ identification of hepatitis C virus RNA in muscle. Neurology.

[8] Chauvin B, Drouot S, Barrail-Tran A, Taburet AM (2013). Drug-drug interactions between HMG-CoA reductase inhibitors (statins) and antiviral protease inhibitors. Clin Pharmacokinet.

[9] Joshi MK, Liu HH (2000). Acute rhabdomyolysis and renal failure in HIV-infected patients: risk factors, presentation, and pathophysiology. AIDS Patient Care STDS.

[10] Douvoyiannis M, Litman N (2009). Acute encephalopathy and multi-organ involvement with rhabdomyolysis during primary HIV infection. Int J Infect Dis.

[11] Towner W, Leyden W, Chao C (2018). Rhabdomyolysis in HIV-infected versus HIV-uninfected persons enrolled in Kaiser Permanente California. https://idsa.confex.com/idsa/2011/webprogram/Paper30490.html.

[12] Chariot P, Ruet E, Authier FJ, Lévy Y, Gherardi R (1994). Acute rhabdomyolysis in patients infected by human immunodeficiency virus. Neurology.

